# Molecular and genomic approach for understanding the gene-environment interaction between Nrf2 deficiency and carcinogenic nickel-induced DNA damage

**DOI:** 10.3892/or.2012.2057

**Published:** 2012-09-26

**Authors:** HYE LIM KIM, YOUNG ROK SEO

**Affiliations:** 1 Department of Life Science, Dongguk University, Jung-gu, Seoul 100-715; 2 Institute of Environmental Medicine for Green Chemistry, Dongguk University, Jung-gu, Seoul 100-715; 3 Department of Pharmacology, Institute for Biomedical Science Institute (IBMS), School of Medicine, Kyung Hee University, Seoul 130-701, Republic of Korea

**Keywords:** nickel, nuclear factor eryrhroid-2 related factor 2, oxidative stress, DNA damage, toxicogenomics, pathway analysis

## Abstract

Nickel (II) is a toxic and carcinogenic metal which induces a redox imbalance following oxidative stress. Nuclear factor erythroid-2 related factor 2 (Nrf2) is a redox factor that regulates oxidation/reduction status and consequently mediates cytoprotective responses against exposure to environmental toxicants. In this study, we investigated the protective roles of the Nrf2 gene against oxidative stress and DNA damage induced by nickel at sub-lethal doses. Under nickel exposure conditions, we detected significantly increased intracellular ROS generation, in addition to higher amounts of DNA damage using comet assay and γ-H2AX immunofluorescence staining in Nrf2 lacking cells, as compared to Nrf2 wild-type cells. In addition, we attempted to identify potential nickel and Nrf2-responsive targets and the relevant pathway. The genomic expression data were analyzed using microarray for the selection of synergistic effect-related genes by Nrf2 knockdown under nickel treatment. In particular, altered expressions of 6 upregulated genes (CAV1, FOSL2, MICA, PIM2, RUNX1 and SLC7A6) and 4 downregulated genes (APLP1, CLSPN, PCAF and PRAME) were confirmed by qRT-PCR. Additionally, using bioinformatics tool, we found that these genes functioned principally in a variety of molecular processes, including oxidative stress response, necrosis, DNA repair and cell survival. Thus, we describe the potential biomarkers regarded as molecular candidates for Nrf2-related cellular protection against nickel exposure. In conclusion, these findings indicate that Nrf2 is an important factor with a protective role in the suppression of mutagenicity and carcinogenicity by environmental nickel exposure in terms of gene-environment interaction.

## Introduction

Nickel (II) has been considered a hazardous heavy metal due to its ability to induce cytotoxicity and carcinogenicity as the result of environmentally persistent exposure. In 1990, the International Agency for Research on Cancer (IARC) classified nickel compounds as group 1 carcinogens (confirmed carcinogen) in humans on the basis of experiments conducted in animals which showed several types of tumors, such as lung and nasal cancers (1). Although the molecular mechanisms of toxicity and carcinogenicity of nickel remain poorly understood, one commonly suggested mechanism for nickel toxicity is the induction of oxidative DNA damage (2). Nickel compounds generate reactive oxygen species (ROS) via a Fenton-type reaction, or via the inactivation of enzyme activities involved in cellular defenses against reactive oxygen systems (3), leading to single and double-strand DNA breaks (4,5).

Intracellular redox homeostasis is maintained by balancing ROS production with ROS removal via cellular antioxidant defense systems (6). The nuclear factor erythroid-2 related factor 2 (Nrf2) is one of the major factors inherent to cellular defenses against oxidative stress (7,8). Particulary, Nrf2-antioxidant response element (ARE) signaling is regarded as a promising strategy in cancer prevention due to its pivotal role in the transcriptional activation of cytoprotective genes facilitating the detoxification of carcinogens (9). Ramos-Gomez *et al*(10) have reported that increased sensitivity to carcinogen and abrogated chemoprotective efficacy of enzyme inducers were found in Nrf2-deficient mice. The Nrf2 cytoprotective adaptive response has evolved as a component of the molecular protection strategies in organisms against exposure to environmental toxicants (11). In our previous study, we have consistently demonstrated the protective effects of Nrf2 on genotoxicity status induced by cadmium treatment in Nrf2-deficient cells (12).

Recently, it has been emphasized the significance of gene-environment interactions in lethal diseases including cancer. A possible definition of gene-environment interaction is that it occurs when a genetic factor and environmental exposure work together to cause a disease outcome in some or all cases. In particular, genetically based variability (silencing, polymorphism) of the carcinogen metabolizing proteins may influence susceptibility to environmental carcinogens (13). Masuko *et al*(14) have reported that the incompetence of Nrf2 might accelerate the development of inflammatory obstructive lung diseases, such as asthma and chronic obstructive pulmonary disease (COPD) after smoking a cigarette containing various heavy metals.

Microarray techniques allow for the simultaneous quantitative analysis of more than one thousand genes, and may prove to be a powerful tool in toxicological studies as well (15,16). In particular, this tool has been applied to evaluate the putative toxicity of environmental pollutants, and established several databases of gene expression patterns induced by toxic chemicals (17). Determining the meaning of the observed biological changes in transcript levels requires the clear elucidation of biological interdependencies. Therefore, it is necessary to analyze the relevant pathways or networks, including significantly regulated genes, in order to explain whole chains of events observed in microarray experiments (18). Several reports have demonstrated that heavy metals as environmental pollutant exhibited a variety of potential toxicity mechanisms, including oxidative stress, disturbances in the homeostasis of essential metals, and interference of interactions with cellular macromolecules (19,20).

In this study, we investigated the protective role of Nrf2 against nickel-induced toxicity in terms of the inhibition of oxidative stress and DNA damage. Additionally, we carried out DNA microarray analyses to profile the gene expression pattern and pathway analysis to identify the specific networks among these genes in environmental pollutant nickel- and/or a Nrf2 gene-specific siRNA-treated human cell line.

## Materials and methods

### Cell culture

RKO (ATCC CRL-2577), human colon cancer cells were cultured in RPMI-1640 (Gibco, Carlsbad, CA, USA), supplemented with 10% fetal bovine serum (FBS) (Gibco) and 1% antibiotics (Gibco) at 37°C in a 5% CO_2_ incubator.

### Transfection of Nrf2-siRNA

RKO cells were plated in 6-well plates at a density of 1×10^5^ cells/well. The next day, the cells were transfected with Nrf2 siRNA using Oligofectamine (Invitrogen GmbH, Karlsruhe, Germany) in accordance with the manufacturer’s recommendation. Specific silencing was confirmed using western blot analysis (data not shown). The Nrf2-targeted siRNA sequence (sense, 5′-GAGUAUGAGCU GGAAAAACdTdT-3′ and antisense: 5′-GUUUUUCCAGC UCAUACUCdTdT-3′) were synthesized by Dharmacon (Thermo Scientific, USA).

### Nickel treatment

Nickel (II) acetate was purchased from Sigma Co. (St. Louis, MO, USA). We determined the sublethal concentration of nickel for our experimental design using the MTT (3-[4,5-dimethyl-2-thiazol-2-yl]-2,5-diphenyltetrazolium bromide) assay and FACS analysis (data not shown). After 24 h of siRNA transfection, cells were treated with low concentration of 20 μM nickel acetate for 12 or 24 h.

### Detection of oxidative stress

In order to assess changes in the intracellular ROS levels as the result of nickel treatment and the absence of Nrf2, we employed an oxidant-sensitive fluorescent probe, 2′,7′-dichlorodihydrofluorescein diacetate (H_2_DCFDA) (Sigma). Non-fluorescent H_2_DCFDA turned into the florescent H_2_DCF as the result of a reaction with intracellular ROS. After heavy metal treatment in either Nrf2 wild-type or Nrf2 siRNA-treated cells, H_2_DCFDA was added at 0.5 μM diluted in PBS and incubated for 30 min at 37°C. The cells were observed in PBS under a Leica DM IRB fluorescence microscope (Wetzlar, Germany).

### Comet assay

The comet assay (single-cell gel electrophoresis) was conducted as described with minor modifications (21). In brief, cells were suspended in 0.5% (w/v) low-melting point agarose, and spread onto microscope slides pre-coated with 1% (w/v) normal-melting point agarose, then covered with a further layer of low-melting agarose. The slides were subsequently dipped into a lysis solution (2.5 M NaCl, 100 mM Na_2_EDTA and 10 mM Tris, pH 10.0) and placed overnight at 4°C in darkness, in order to solublize the cell membranes and cytoplasm. Upon completion of lysis, the slides were placed in a horizontal gel electrophoresis tank with alkaline electrophoresis buffer (300 mM NaOH and 1 mM Na_2_EDTA, pH 13.0) and left in the solution for 20 min at 4°C to allow the DNA to unwind and the alkali-labile sites to express. Electrophoresis was conducted for 15 min at 25 V and 250 mA. After being run, slides were neutralized with neutralization buffer and washed in PBS. The slides were air-dried at room temperature. Then, the slides were stained with SYBR Gold solution and observed under a fluorescence microscope (Nikon Eclipse 50i, Nikon, Japan). One hundred cells were scored for DNA damage intensity at each experiment point from two different slides and the means were calculated.

### γ-H2AX immunofluorescence staining

The formation of nuclear foci containing phosphorylated histone H2AX was assessed by immunofluorescence. Briefly, either RKO or Nrf2 knockdown cells grown on glass coverslip were treated without and with nickel for 24 h, followed by incubation in selective fresh media for additional 12 h. The cells were fixed with iced methanol for 30 min at −20°C and rinsed with iced acetone for a few seconds. After washing with PBS, the cells were incubated with an anti-γ-H2AX primary antibody (Active Motif, Inc., Carlsbad, CA, USA) overnight at 4°C and an anti-rabbit-Cy3 secondary antibody (Jackson ImmunoResearch, USA) for 1 h at room temperature. After PBS washing, the cells were mounted with mounting solution containing DAPI nuclear stain. The coverslips were placed onto slides and the foci were visualized under fluorescence microscope (Nikon, Japan).

### Total RNA preparation for microarray experiment

To isolate total RNA, frozen cell pellets were homogenized in lysis buffer containing 2-mercaptoethanol. Total RNA was subsequently purified using RNeasy kits (Qiagen, Valencia, CA, USA) in accordance with the manufacturer’s recommendations, prior to the microarray experiments.

### Focused DNA microarray and data analysis

The genes involved in DNA damage, DNA repair, apoptosis, oxidative stress, and cell cycle were selected to design the focused microarray chip. The information of all genes was found in the UniGene Build 189 and Agilent databases (http://earray.chem.agilent.com). Transcriptomic studies were conducted using an 8*15K Oligo chip (Agilent) and a Quick Amp Labeling kit in accordance with the manufacturer’s protocols. Subio platform ver. 1.6 was used for the initial analysis of expression data. Expression changes are described as fold-changes (expression ratio between control- and treated-signals) (22).

### Real-time quantitative RT-PCR (qRT-PCR)

Real-time qRT-PCR was performed using Power SYBR-Green PCR kit (Applied Biosystems, Carlsbad, CA, USA) according to the manufacturer’s instructions. The primers of 10 genes analyzed from microarray data were designed for qRT-PCR (Table III). Thermal cycling conditions were undertaken as follows: 50°C for 2 min and 95°C for 10 min followed by 40 cycles of 95°C for 30 sec and 60°C for 30 sec, 72°C for 30 sec. The real-time PCR analysis was performed on an Applied Biosystems Prism 7900HT Sequence Detection System (PE Applied Biosystems).

### Pathway analysis

We used Pathway Studio 7.1 software (Ariadne Genomics, Rockville, MD, USA) to define the cellular networks and interactions among genes expressed in our microarray experiment. This software contains >100,000 regulations, interactions, modifications and cell process events between proteins and small molecules. The database has been compiled by the application of the text-mining tool MedScan to the entirety of PubMed (23). Additionally, Pathway Studio enables the visualization of gene expression values and status in the context of protein interaction networks and pathways.

## Results

### ROS level was increased in Nrf2 knockdown cells in response to nickel

**Figure 1:**
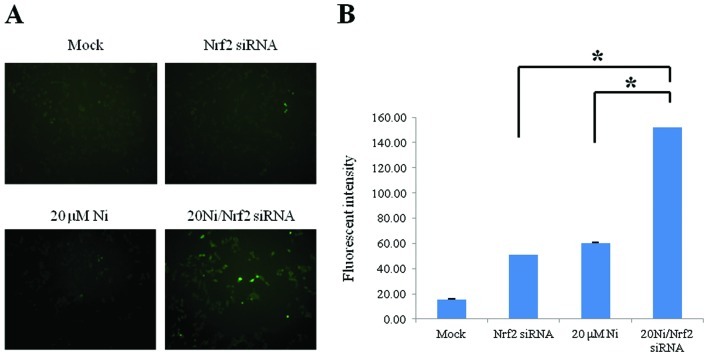
The measurement of oxidative stress in nickel-treated Nrf2 lacking RKO cells. (A) Intracellular ROS were detected using 2′,7′-dichlorodihydrofluorescein diacetate (H_2_DCHDA). The levels of ROS were increased in Nrf2 knockdown RKO cells under 20 μM nickel exposure, in comparison to wild-type cells. (B) The average of fluorescence intensity in the H_2_DCHDA-stained cells was quantified. The asterisks indicate significant difference at P-value <0.05.

Nrf2 as a redox factor has a vital role in protection by regulating the cellular oxidation/reduction status. To evaluate the protective roles of Nrf2 against oxidative stress induced by a sub-lethal 20 μM nickel exposure (data not shown), we constructed a transient Nrf2 knockdown system in human RKO cells via siRNA transfection (data not shown). The level of oxidative stress was assessed via immunofluorescence detection using ROS-sensitive H2DCHDA. We observed significantly increased intracellular ROS levels in the nickel-treated Nrf2 lacking cells than that in the nickel-treated wild-type cells (Fig. 1). This result indicates that Nrf2 might be an important redox modulator to suppress nickel-induced oxidative stress.

### The amount of DNA strand breaks were significantly augmented in nickel-treated Nrf2-silencing cells

**Figure 2:**
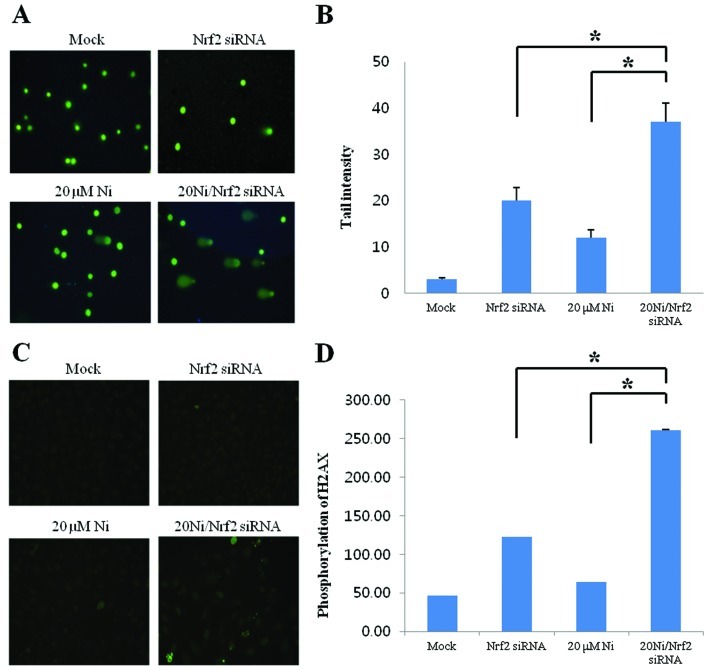
The detection of DNA strand break in nickel-treated Nrf2 lacking RKO cells. (A) The alkaline comet assay was conducted to assess DNA damage in wild-type and Nrf2 knockdown cells under nickel treatment. Nickel exposure in the Nrf2 knockdown cells induced significantly high levels of DNA strand breaks as compared to the wild-type cells. (B) Comet tail length was measured in arbitrary units. (C) The DNA strand breaks were detected by γ-H2AX immunofluorescence staining in wild-type and Nrf2 knockdown cells under nickel exposure. In Nrf2 knockdown cells, the higher amount of foci were found in response to nickel. (D) Fluorescence density of foci was analyzed with a microscope. The asterisks indicate significant difference at P-value <0.05.

Nickel exposure induces ROS with the subsequent generation of oxidative DNA damage (2). To evaluate the functions of Nrf2 in inhibition of nickel-induced DNA damage, we employed the comet assay and γ-H2AX immunofluorescence staining to detect DNA strand breaks as indicator of oxidative DNA damage. In Fig. 2, we demonstrated a significant increase in damaged DNA in Nrf2-lacking RKO compared to wild-type cells under nickel treatment, thereby indicating the critical protective role of Nrf2 factor against nickel-caused DNA damage.

### The potential nickel- and Nrf2-target genes were discovered using toxicogenomic tool

**Figure 3:**
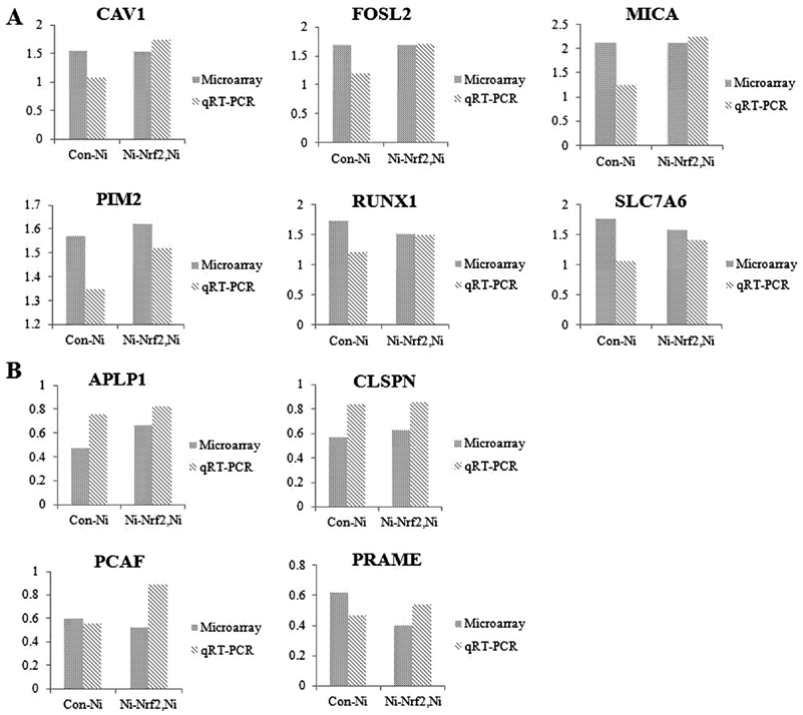
Comparison of gene expression patterns obtained from DNA microarray and qRT-PCR in Nrf2 knockdown under nickel exposure. Bars indicate the magnitude of gene expression changes in DNA microarray and qRT-PCR, respectively. Y-axis denotes the fold expression change of the respective gene. (A and B) The expressions of the upregulated and downregulated genes, respectively.

The alteration of genomic expression patterns against nickel depending on functional Nrf2 status was analyzed using focused DNA microarray experiments. Microarray experiments were conducted after 24 h of nickel treatment at sub-lethal concentration in Nrf2 knockdown cells compared to wild-type cells. As a results of the toxicogenomics assays, we detected a total of 17 genes that were significantly altered by Nrf2 knockdown under nickel exposure condition (>1.5-fold and P<0.05). Six notable genes were upregulated (Table I) and 11 genes were downregulated (Table II). Additionally, we demonstrated that the expression levels from the microarray results of 10 genes (CAV1, FOSL2, MICA, PIM2, RUNX1, SLC7A6, APLP1, CLSPN, PCAF and PRAME) were consistent with those shown in the qRT-PCR experiment (Fig. 3). These data implied that these validated genes might be regarded as potential markers for understanding the interaction of Nrf2-oriented genes in response to nickel-induced toxicity.

### Nrf2-responsive genes under nickel exposure were involved in toxicity-prone and cytoprotective pathway

**Figure 4:**
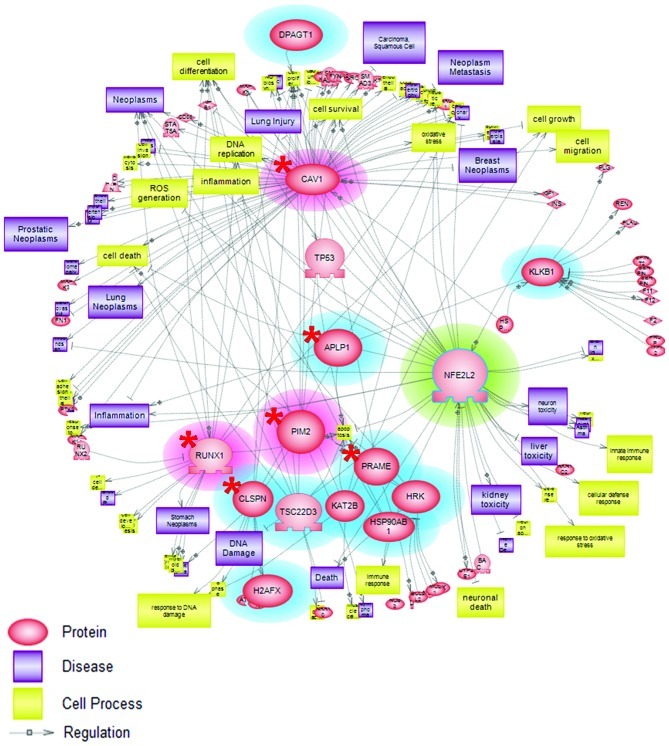
Pathway analysis of toxicogenomic data. Magnified red circles indicate upregulated genes and blue circles indicate downregulated genes in our microarray data. Genes validated by qRT-PCR are indicated by asterisks. The bioinformatics analysis of our microarray data using Pathway Studio software (version 7.1) show nickel and Nrf2 responsive relationships in terms of cell processes and diseases.

Networks are used ubiquitously throughout biology to represent the relationships between genes and gene products. Pathway Studio software is capable of establishing a more dynamic model of cellular processes via the incorporation of gene expression data. We employed Pathway Studio (version 7.1) to evaluate possible interactions among Nrf2- and nickel-responsive genes obtained from our microarray data analysis. Our analyzed results showed that potential nickel- and Nrf2-target genes from microarray data were mainly associated with oxidative stress, inflammation, apoptosis, cell cycle and cell survival processes (Fig. 4). These results provide insights into the manner in which these upregulated and downregulated genes were critical in mediating toxicity-prone responses and protection of the cells.

## Discussion

The toxicity of nickel has become a matter of some interest because of the widespread environmental release of nickel and the incidence of accidental poisoning in nickel workers (24). One possible mechanism underlying the toxicity and carcinogenicity of nickel compounds in humans and animals evolves the induction of DNA damage related to oxidative stress. The Nrf2-regulated signaling pathway plays an important role in protection against oxidative stress-mediated cytotoxicity after exposure to oxidants. Increased oxidative stress occurring as the result of the destruction of redox homeostasis has been implicated in the etiology of a number of acute and chronic diseases linked to exposures to environmental toxicants such as heavy metals. In this study, we investigated the protective roles of Nrf2 against nickel-induced toxicity at sub-lethal dose in terms of the suppression of oxidative stress and DNA damage. Additionally, we suggested the cytotoxic and protective cellular mechanisms among the selected nickel and Nrf2-related genes using microarray analysis.

In general, nickel exposure causes accumulation in human at low concentrations over the long-term. Therefore, we selected 20 μM of nickel as a sub-lethal dose, and detected high cell survival rates and unchanged cell cycles (data not shown). Our data showed that under nickel treatment, Nrf2 siRNA-treated cells produced higher levels of ROS and DNA strand breakage, in comparison to Nrf2 wild-type cells (Figs. 1 and 2). Nickel enhanced oxidative stress in plasma by stimulating the generation of ROS, including ^•^OH, O2^•−^, H_2_O_2_(25). Gong and Cederbaum (26) have previously shown that the knockdown of Nrf2 in a human liver cell line caused an increase in ROS levels and a reduced expression of antioxidative enzymes. Several reports have mentioned the protective effects of Nrf2 in response to various environmental toxic chemicals. Aoki *et al*(27) showed an increase in the levels of 8-oxodeoxyguanosine (8-oxo-dG) in bronchial epithelial cells from Nrf2 knockout mice compared to wild-type sub-chronically exposed to diesel exhaust particles, thereby indicating that the absence of Nrf2 could contribute to an increase in oxidative DNA damage and adduct formation. Additionally, our previous study has demonstrated an increased level of DNA strand breaks, following the induction of oxidative stress in Nrf2 siRNA-treated cells under carcinogenic metal cadmium exposure conditions (12).

We can explain these toxic molecular mechanisms by analyzing differentially expressed genes using microarray and qRT-PCR. In our study, six genes (CAV1, FOSL2, MICA, PIM2, RUNX1 and SLC7A6) were notably upregulated in nickel-treated Nrf2 knockdown cells as compared to nickel-treated wild-type cells (Fig. 3A and Table I). Caveolin 1 (CAV1) and FOS-like antigen 2 (FOSL2) expressions have been shown to be elevated in many human cancer cell lines and numerous human tumor specimens (28,29), thereby indicating that CAV1 and FOSL2 play important roles in the promotion of mammary tumorigenesis. Particularly, these genes have been associated with the development and progression of breast cancer (30,31). MHC class I polypeptide-related sequence A (MICA) is overexpressed as the result of a DNA damage response involving the ATM (ataxia telangiectasia mutated) and the ATR (ATM- and Rad3-related) protein kinases (32). Del Toro-Arreola *et al*(33) have also reported that MICA was strongly upregulated after genotoxic stress in human cervical cancer cell lines. The Pim-2 (PIM2) is a novel oncogene and its protein has a profound inhibitory effect on the apoptosis of tumor cells without cell specificity (34). PIM2 and runt-related transcription factor 1 (RUNX1) oncogenes have critical role in the development of several kinds of tumors, including prostatic carcinoma (35,36). Additionally, the expression of LAT family members, including the solute carrier family 7, member 6 (SLC7A6; LAT3) gene has been observed in a variety of malignant cells (37,38). Pathway analysis results using Pathway Studio software (version 7.1) demonstrated that upregulated genes observed by microarray analysis were involved in the regulation of gene expression associated with oxidative stress, inflammation, and necrosis processes (Fig. 4). Hereby, we might postulate that CAV1, FOSL2, MICA, PIM2, RUNX1 and SLC7A6 genes could be regarded as potential targets of nickel and Nrf2-regulated genes.

We also discovered downregulated genes in response to nickel and redox status. The expressions of APLP1, CLSPN, PCAF and PRAME were downregulated in nickel and Nrf2 siRNA-treated cells as compared to cells treated only with nickel (Fig. 3B and Table II). Amyloid-β precursor-like protein (APLP1) is a member of the amyloid precursor protein (APP) family, harboring the copper (Cu) binding domain. APP modulates the toxic reactive oxygen intermediates generated by unregulated redox reactivity of Cu (39). Tang *et al*(40) have reported that APLP1 might be a novel transcriptional target of p53, according to the results of *in vivo* and *in vitro* characterization of a p53 responsive element found in the first intron of the APLP1 gene locus. Similarly, p300/CBP-associated factor (PCAF) has been shown to acetylate p53 in response to DNA damage, resulting in the increased transcription of p53-regulated genes (41). Additionally, the claspin homolog (CLSPN) is an essential upstream regulator of checkpoint kinase 1 and triggers a checkpoint arrest of the cell cycle in response to replicative stress or DNA damage (42). The gene is also required for efficient DNA repair system (43) and DNA replication during a normal S phase (44). The preferentially expressed antigen of melanoma (PRAME) gene is involved in the regulation of cell death or cell cycle. Furthermore, this gene induces caspase-independent cell death *in vitro* and reduces tumorigenicity *in vivo*(45). From pathway results, downregulated genes were shown to mediate cell cycle arrest, and cell survival (Fig. 4). These results implied that the abovementioned downregulated genes might be possible markers useful for assessing the relationship between nickel and the Nrf2 gene.

**Figure 5:**
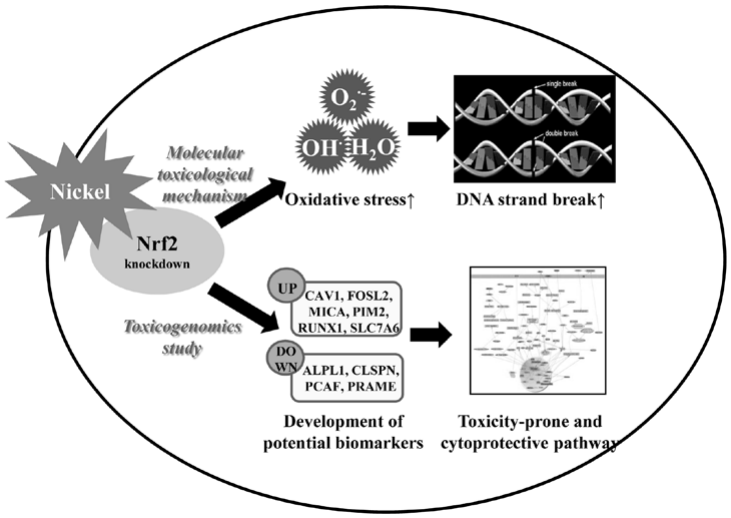
Scheme showing synergistic genotoxic effects between Nrf2 knockdown and nickel exposure. Nickel can stimulate the intracellular increase of ROS generation and DNA strand break in Nrf2 knockdown cells, as compared to Nrf2 wild-type cells. In addition, we suggest the potential biomarkers and molecular pathway for Nrf2-related cellular protection against nickel exposure. Our results emphasize the significance of the study of interaction between genes and environments.

In summary, we demonstrated the synergistic toxic effects of Nrf2 gene knockdown following exposure to the environmental toxicant nickel, in terms of gene-environment interaction (Fig. 5). Nrf2 gene silencing accelerated an increase in nickel-induced oxidative stress and DNA damage. Using microarray and qRT-PCR, we found several genes (CAV1, FOSL2, MICA, PIM2, RUNX1, SLC7A6, APLP1, CLSPN, PCAF and PRAME) markedly enhanced or reduced expression levels in nickel-exposed Nrf2 knockdown cells as compared to nickel-treated wild-type cells. These up- and downregulated genes perform a crucial function in toxicity-prone and cytoprotection, respectively. Thereby, the nickel and Nrf2-responsive genes detected in our study might be useful in the development of strategies targeting the Nrf2 pathway for the purposes of attenuating environmental diseases, including cancer.

## Figures and Tables

**Table I:** List of 1.5-fold upregulated genes in response to nickel exposure in Nrf2 knockdown cells.

Gene symbol	Accession no.	Gene name	Con-Ni^a^		Ni-Nrf2,Ni^b^	
	
			Fold-change	P-value	Fold-change	P-value
CAV1	NM_001753	Caveolin 1, caveolae protein, 22 kDa	1.54	0.000167	1.52	0.00015
FOSL2	NM_005253	FOS-like antigen 2	1.68	0.00235	1.68	0.00289
MICA	NM_000247	MHC classIpolypeptide-related sequence A	2.13	0.0000063	2.13	0.00000368
PIM2	NM_006875	Pim-2 oncogene	1.57	0.00000675	1.62	0.00165
RUNX1	NM_001001890	Runt-related transcription factor 1 (acute myeloid leukemia 1; aml1 oncogene)	1.73	0.000165	1.51	0.00239
SLC7A6	NM_001076785	Solute carrier family 7 (cationic amino acid transporter, y+ system), member 6	1.77	0.0000165	1.58	0.00011

a Con-Ni indicates the ratio between expression level in the nickel-treated sample compared to the wild-type sample.

b Ni-Nrf2,Ni indicates the ratio between expression level in the nickel-treated Nrf2-knockdown sample compared to the nickel-treated wild-type sample.

**Table II:** List of 1.5-fold downregulated genes in response to nickel exposure in Nrf2 knockdown cells.

Gene symbol	Accession no.	Gene name	Con-Ni^a^		Ni-Nrf2,Ni^b^	
	
			Fold-change	P-value	Fold-change	P-value
APLP1	NM_005166	Amlyoid β (A4) precursor-like protein 1	0.47	0.00101	0.66	0.000268
CLSPN	NM_022111	Claspin homolog (Xenopus laevis)	0.57	0.00278	0.62	0.04623
FANCB	NM_001018113	Fanconi anemia, complementation group B	0.64	0.00136	0.58	0.03481
H2AFX	NM_002105	H2A histone family, member X	0.35	0.0000521	0.6	0.000443
HRK	NM_003806	Hara-kiri, BCL2 interacting protein (contains only BH3 domain)	0.35	0.000758	0.59	0.00671
HSP90AB1	NM_007355	Heat shock protein 90kDa α (cytosolic), class B member 1	0.51	0.00213	0.53	0.00998
KLK3	AF335478	Kallikrein-related peptidase 3	0.2	0.0000151	0.55	0.00176
PCAF	NM_003884	P300/CBP-associated factor	0.6	0.00606	0.52	0.0128
PRAME	NM_206956	Preferentially expressed antigen in melanoma	0.62	0.00456	0.4	0.000403
RBMX	NM_002139	RNA binding motif protein, X-linked	0.65	0.00641	0.4	0.00971
TSC22D3	NM_004089	TSC22 domain family, member 3	0.47	0.00277	0.53	0.00855

a Con-Ni indicates the ratio between expression level in the nickel-treated sample compared to the wild-type sample.

b Ni-Nrf2,Ni indicates the ratio between expression level in the nickel-treated Nrf2-knockdown sample compared to the nickel-treated wild-type sample.

**Table III:** List of gene-specific primer sequences used in the qRT-PCR validation study.

Gene name	Forward	Reverse
CAV1	TCT CTA CAC CGT TCC CAT CC	ACT TGC TTC TCG CTC AGC TC
FOSL2	AGC CTT GGA GAA CTC GGT TT	AAC AAA GGG ACA GGA ATG GTC
MICA	TTC CAT GTT TCT GCT GTT GC	ACT GGG TGT TGA TCC AGG AC
PIM2	GGA ATG GAA GAT GGA CAC CA	AAA CAG CAA GCC TTA TTT CCC
RUNX1	GGA TCT CGC TGT AGG TCA GG	CTC CGG GAA TCT TCC TGT TT
SLC7A6	TGC TCT GGC CTA ATG GAT CT	GAC TGC CTC ACT GTT GAC CA
APLP1	GCC TGC CTG GTG AAT TTG TG	GCC TCC GGG TTG AAC TCT C
CLSPN	ATG ATT CCC AGA TGG ACT TG	AGC CAC TGC TCT CGT TCA AT
PCAF	AAA GAT GGC CGT GTT ATT GG	CCA TAG CCC TTG ACT TGC TC
PRAME	ATG GAA CGA AGG CGT TTG TG	GTG TCT CCC GTC AAA GGC T
